# Strategies for Rational Use of Personal Protective Equipment (PPE) Among Healthcare Providers During the COVID-19 Crisis

**DOI:** 10.7759/cureus.8248

**Published:** 2020-05-23

**Authors:** Syed Uzair Mahmood, Faine Crimbly, Sheharyar Khan, Erum Choudry, Syeda Mehwish

**Affiliations:** 1 Pediatrics, The Indus Hospital, Indus Hospital Research Center, Karachi, PAK; 2 Pediatrics, Jinnah Sindh Medical University, Karachi, PAK; 3 Family Medicine, Baqai Medical University, Karachi, PAK; 4 Dentistry, The Indus Hospital, Indus Hospital Research Center, Karachi, PAK; 5 Dentistry, Karachi Medical and Dental College, Karachi, PAK

**Keywords:** ppe shortage, ppe strategies, healthcare worker safety, personal protective equipment (ppe), coronavirus 2019 (covid-19)

## Abstract

As the coronavirus 2019 (COVID-19) began spreading globally with no clear treatment in sight, prevention became a major part of controlling the disease and its effects. COVID-19 spreads from the aerosols of an infected individual whether they are showing any symptoms or not. Therefore, it becomes nearly impossible to point exactly where the patient is. This is where personal protective equipment (PPE) comes in. These are masks, respirators, gloves, and in hospitals where the contact with the infected and confirmed patient is direct, also gowns or body covers. The PPEs play a major role in the prevention and control of the COVID-19. The PPE is able to prevent any invasion of the virus particles into the system of an individual which is why it is an essential item to have for healthcare workers. Due to the high demand for PPEs all around the world, it is important to optimize the use of protective gear and ration the supplies so that the demand are met. However, there are guidelines recommended by the World Health Organization (WHO) and the Centers for Disease Control and Prevention (CDC) to maintain the supply in the wake of this increased demand of PPE, how the manufacturers should track their supplies, and how the recipients should manage them. Various strategies can be used to increase the re-use of PPEs during the COVID-19 pandemic that has modified the donning and doffing procedure.

## Introduction and background

**Figure 1:**
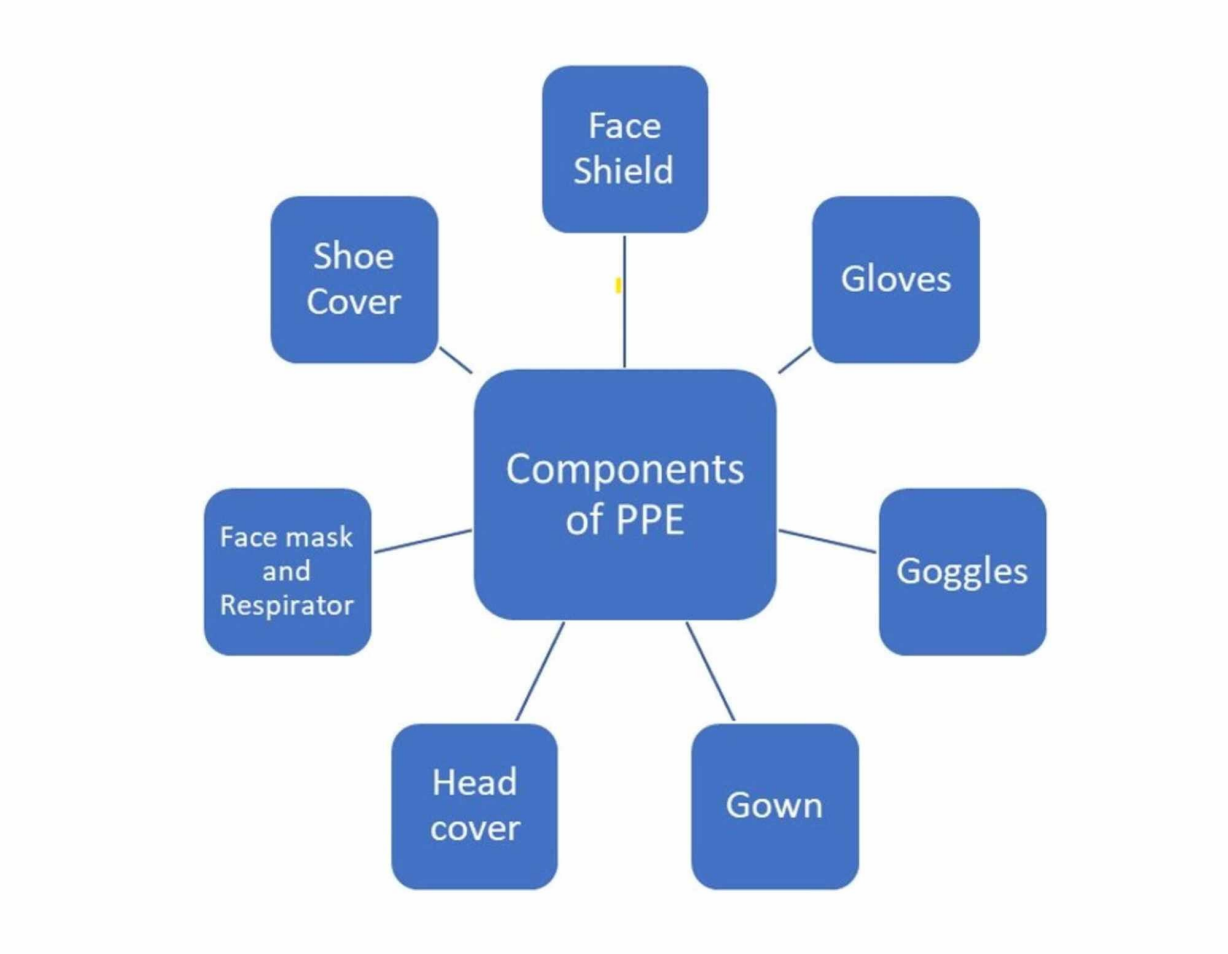
Components of personal protective equipment (PPE)

Personal protective equipment (PPE) is an article used to prevent the wearer from coming in contact with hazardous, infectious, chemical, radiological, electrical, and physical agents. It contains components illustrated in Figure 1 [1]. The surge in demand and misuse of PPE has led to an acute shortage of protective gear, endangering the lives of healthcare workers [2]. More than 9,000 healthcare workers (HCW) in the United States (US) and more than 17,000 in Italy have been infected with COVID-19 [3-4]. A total of 444 HCW in Pakistan have been exposed to COVID-19 as of April 29, 2020 [5]. Many doctors are performing their duty without any PPE and are at high risk of becoming infected [6]. There have been peaceful protests all over the world by doctors, nurses, and other healthcare professionals demanding PPE.

In the wake of the COVID-19 pandemic, PPE plays a significant role, with face masks and gloves being the most essential. Doctors, nurses, and other frontline healthcare responders are using them to minimize the risk of contaminated contact or droplet exposure. Some studies suggest that the psychological impact of PPE is such that individuals using them might feel more protected than they actually are in reality [7]. It should be ensured that the wearer practices hand hygiene before wearing and after removing the protective gear. Also, an appropriate method for its disposal should be considered.

## Review

Is wearing PPE important?

The primary mode of transmission of coronavirus is known to be droplet or contact-based. Infected individuals are prone to spread the virus while coughing, sneezing, or speaking. This micro virus, when ejected, can travel up to a distance of six feet. Wearing a face mask, along with other precautionary measures like hand hygiene and self-isolation, limits the transmission of infectious agents [8-9].

Initially, the usage of masks among the general public was highly controversial. Experts discouraged healthy people from wearing masks due to the scarce supply. This equipment was reserved for those in direct contact with infected patients [10]. However, the rapid rise in the degree of local transmission has caused many countries to allow their citizens to wear non-medical/cloth masks, along with practicing social distancing [9, 11]. Evidence-based studies reveal that the concomitant use of household (non-medical) face masks, as well as using a proper handwashing regimen, reduces the probability of local transmission, thereby decreasing the death toll [12]. It should be noted that according to the World Health Organization (WHO) guidelines, medical masks and respirators should only be reserved for healthcare workers [10]. Factors that determine the efficiency of face masks are listed in Table 1 [13].

**Table 1:** Factors That Determine the Efficiency of Masks

Factors that determine the efficiency of masks
Number of layers
The shape of the mask
Facial fit
Breathing ability of the material used
Filtration efficiency

Types of masks

The main types of masks being used are respirators, medical masks, and non-medical/cloth masks. 

Respirators

These are protective equipment which provides an almost accurate facial fit and effective filtration of airborne particles. They provide a proper seal around the mouth and nose, providing optimal protection. According to the recent WHO, CDC, and FDA guidelines, such masks are only reserved for healthcare providers [10, 13-14]. The FDA has labeled these masks as single-use, disposable devices; however, in cases of shortage in supplies, these can be sterilized and reused [14]. While the respirator masks are highly efficient, they still do not provide complete protection. Improper and misuse of these masks can lead to the spread of infection in the user [15].

Medical Masks

These are thin, pleated, and disposable masks that protect the user from inhaling dust particles, contaminated liquid droplets, and bacteria. They are usually two layers thick and made from unwoven fabric. These masks only act as a physical barrier between the user's nose and mouth and the infected environment. They do not possess a proper seal and are less effective than respirators. These are loose masks, which allow comfortable breathing and reduce transmission probability [14].

Non-Medical Masks

According to recent studies, asymptomatic and pre-symptomatic carriers of the novel coronavirus have been detected and can transmit the virus. In the face of this discovery, CDC experts recommend that the general public uses non-medical/cloth coverings to shield their mouth and nose. These textile masks are made up of layers of cloth. Some of them also possess a paper towel layer, which increases the filtration capability. They do not offer full protection but, along with other precautionary measures, are useful to slow down the spread of coronavirus [16].

PPE per clinical setting

As a general safety precaution, every frontline healthcare worker (HCW) should know which PPE needs to be used in different clinical settings [17-18].

1) Under any clinical setting where there is a risk of getting infected, the individual should don (put on) a medical face mask, gloves, gown, and eye protection,

2) If the HCW is more than 2 meters away from the patient, he/she should use a fluid-resistant medical face mask with or without eye and face protection, depending on whether there is exposure to flashes or droplets.

3) In case of an ongoing aerosol-generating procedure (AGP), all individuals present should wear a respirator, face and eye protection, gloves, and long-sleeved fluid-repellent gown.

4) Contact transmission can be avoided by wearing disposable gloves and gowns.

Donning and doffing PPE for infection prevention

**Figure 2:**
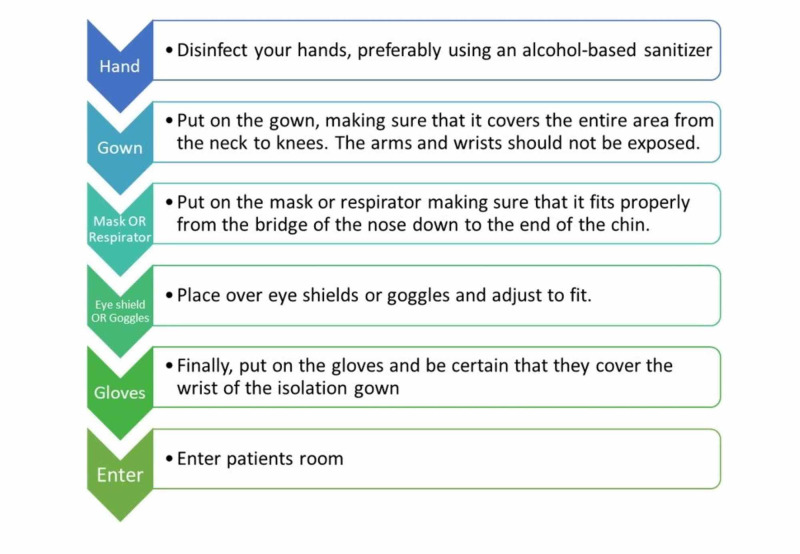
Donning PPE for infection prevention

**Figure 3:**
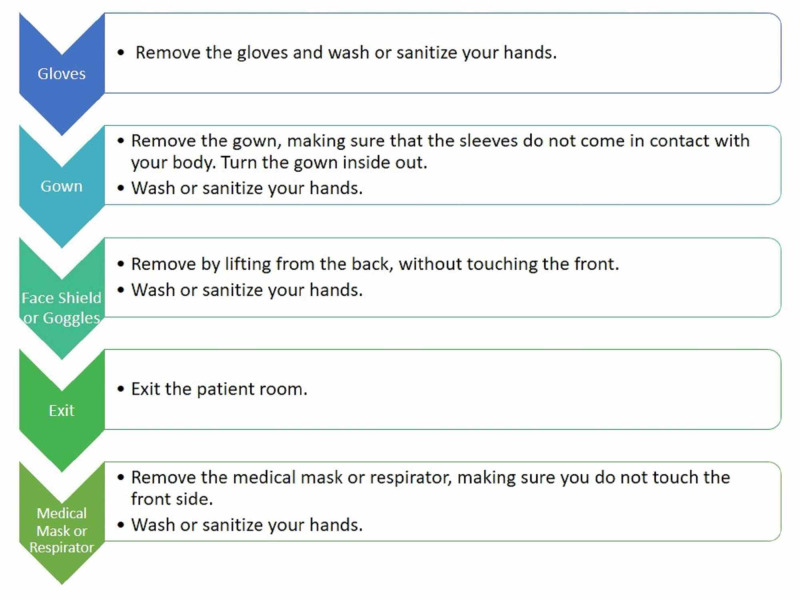
Doffing PPE for infection prevention Note: All PPE should be removed inside the patient's room except for the respirator.

It is essential that every HCW should know the proper way to put on (donning) and remove (doffing) PPE. Any mistake in doing so can render the individual exposed to infections agents. Centers for Disease Control and Prevention (CDC) and the Ministry of Health, New Zealand have introduced the proper way of handling PPE [19-20]. HCW should wear the entire PPE before entering a patient’s room. Steps of donning and doffing are illustrated in Figures 2-3.

Decontamination and reuse of PPE

According to standard Infection Prevention and Control (IPC) guidelines, PPE is a single-use, disposable item. However, due to the current shortage of PPE, health care providers are challenged to rationally use the limited supplies by decontaminating and reprocessing them. It should be noted that there is no proven effectiveness of these practices and priority is given to the rapid manufacture of protective items [21]. Improper or inadequate decontamination of equipment before reuse is unsafe and can pose serious threats [22]. When disinfecting PPE, it is important to keep in mind the efficacy of the method used, check for any residual toxicity, and make sure that the functional integrity of the material is maintained.

General strategies include following the manufacturer’s guidelines to disinfect and reprocess the PPE. Routine inspection of protective material should be carried out, along with the replacement of the equipment if the integrity is not maintained or it is damaged.

Respirators and Medical Masks

1) Usually cleaning prior to disinfection is required. Respirators and medical masks lose their protective property when they undergo cleaning.

2) Considering the current conditions, these items can be worn by a single HCW for multiple shifts. Factors, such as humidity and shelf-life, limit their use.

3) Medical masks can be reprocessed using the Environment Protection Agency (EPA)-registered disinfectants.

4) Filtering facepiece respirators can be decontaminated using vaporous hydrogen peroxide, moist heat, and bleach solution.

5) Remember to replace these respirators if breathing is hindered.

Gowns

1) Submerge in hot water and detergent, then thoroughly scrub the gown.

2) Afterward, soak in 0.05% chlorine solution for about 30 minutes.

3) Rinse in clean water and ideally allow drying in the sun.

4) Gowns having small holes and tears could be mended whereas worn out gowns should be discarded.

Disposable Face Shields

1) Clean first the inside and then the outside surface of the visor using a detergent-soaked clean cloth.

2) Clean the outside of the visor with a clean cloth saturated with disinfectant.

3) Wipe the outside of the visor with clean water.

4) Use towels or dry air to completely dry the visor.

Reusable Goggles and Face Shields

1) Immerse in warm water and neutral detergent solution.

2) Rinse with clean water.

3) Wipe with disinfectant and then again rinse with clean water.

4) Dry completely using towels or dry air.

Proper disposal of PPE

Potentially infectious medical waste (PIMW), such as COVID 19 testing kits and PPE, have a serious risk of coming in contact with infectious bodily fluids. These materials should be kept safely on site (hospitals, testing centers) in secure containers. They should then transferred to storage facilities, where they are disinfected and disposed of off to landfill sites [23]. Individuals responsible for waste management should take caution and should wear appropriate gear. It is extremely critical to properly decontaminate and dispose of any waste material that could infect people who come in contact with it.

PPE crisis strategy

The escalating demand for PPE has given rise to new state and local strategies to ensure the careful optimization of available resources. This policy helps reserve the reduced amount of PPE for the most critical conditions. As the situation improves and the PPE supply is sufficient again, the state can return to its conventional PPE guidelines. The following strategies should be observed to overcome the shortage of PPE [24].

Appropriate use of PPE

There is a difference in the demand and supply of PPE, with severe shortages in supply on all fronts. It is crucial that all the equipment is used with care to prevent wastage, to ensure a continuous supply of protective equipment despite limited production [1, 22].

1) The healthcare professionals who are working with patients of COVID-19 and are in direct contact should have PPE consisting of gloves, gowns, masks, face shields, and goggles.

2) The same respirator can be used while examining multiple patients at a time. Since the shortage of supply is a fact in most places, it is recommended to keep wearing a single one for multiple patients than to not have any respirator on.

3) HCW performing or assisting with invasive procedures should be wearing respirators, eye protection (like goggles), and a face shield aligned with the gown and gloves. If the gowns allow fluid to pass through, an additional layer of protective coverage like an apron should be worn.

4) People who are taking care of the sick at home should be provided with medical masks at home for their own protection and to limit the spread of the disease.

5) Individuals who remain asymptomatic or do not show any signs of illness can use non-medical masks and should not opt for medical masks. Inappropriate use of medical masks may increase the demand and can also impede the supply to professionals who need them the most.

Limit the need for PPE

The need for PPE can be minimized by the following interventions [25]:

1) Limit patient contact and use alternate tools, such as telemedicine, for non-emergency cases.

2) Make sure that no personnel who is not immediately needed for the patients' care should enter the premise of the COVID-19 ward that should be a separated and isolated area. The visitors should either not be allowed at all or should have minimal contact with the patients.

3) All non-urgent procedures/appointments should be postponed.

4) PPE should be reused with proper decontamination guidelines.

5) PPE should be used beyond their shelf life making sure they are not worn out or damaged.

6) In the case of the absolute absence of PPE, alternate methods for barrier control (e.g., glass shields) should be employed.

These practices do not guarantee the absolute safety of healthcare professionals, and their effectiveness is questionable. However, under the present circumstances, these crisis strategies given by the CDC should be duly addressed.

Coordination between the supply and demand of PPE

The supply should be monitored and demand adjusted [21, 26]. This can be done using the following methods:

1) Use of rational quantification-based forecasts regarding PPE. This helps in rationing available supplies to meet the demand.

2) The request for PPE from countries, as well as major responders, should be monitored and controlled. The distribution of PPE to healthcare institutions should be controlled and monitored.

3) To avoid stock duplication, a centralized request management system should be applied that takes notice of whether the stock management rules are being followed or not. This helps in controlling the wastage and overstock.

4) Keep a check on the end-to-end distribution of PPE.

## Conclusions

Due to the recent ease in lockdown measures and the commencement of the holy month of Ramadan in the Muslim world, an abrupt rise in public gatherings is feared. Therefore, it is highly critical that PPE’s should be used in all clinical and non-clinical settings. Citizens should use a cloth barrier while stepping out of the house and public gatherings should be strictly avoided. The proper protocol should be followed when healthcare professionals consider reusing PPE.

As Pakistan is one of the major distributors of PPE throughout the world, it has set an exemplary approach during this pandemic. The Pakistani Government and National Disaster Management Authority (NDMA) have made tireless efforts to increase the manufacturing and distribution of PPE. Moreover, many non-governmental organizations (NGOs) and medical students have come forward to combat this deadly disaster and distribute PPE to those fighting on the frontline.
